# Treatment of Four Stored-Grain Pests with Thiamethoxam plus Chlorantraniliprole: Enhanced Impact on Different Types of Grain Commodities and Surfaces

**DOI:** 10.3390/insects14070619

**Published:** 2023-07-10

**Authors:** Waqas Wakil, Nickolas G. Kavallieratos, Nikoleta Eleftheriadou, Muhammad Sami Ullah, Aqsa Naeem, Khawaja G. Rasool, Mureed Husain, Abdulrahman S. Aldawood

**Affiliations:** 1 Department of Entomology, University of Agriculture, Faisalabad 38040, Pakistan; samimuhammad103@gmail.com (M.S.U.); aqsanaeem231@gmail.com (A.N.); 2 Senckenberg German Entomological Institute, D-15374 Müncheberg, Germany; 3 Laboratory of Agricultural Zoology and Entomology, Department of Crop Science, Agricultural University of Athens, 75 Iera Odos Str., 11855 Athens, Greece; nikolelef@aua.gr; 4 Department of Plant Protection, College of Food and Agriculture Sciences, King Saud University, P.O. Box 2460, Riyadh 11451, Saudi Arabia; gkhawaja@ksu.edu.sa (K.G.R.); mbukhsh@ksu.edu.sa (M.H.); aldawood@ksu.edu.sa (A.S.A.)

**Keywords:** neonicotinoid, anthranilic diamide, mortality, progeny, persistence, pest management

## Abstract

**Simple Summary:**

Among pests threatening stored food, *Rhyzopertha dominica*, *Tribolium castaneum*, *Trogoderma granarium*, and *Sitophilus oryzae* are highly dangerous because they cause considerable losses around the world. Taking into account that there is a narrow available spectrum of contact insecticides for the management of stored-grain insects, a laboratory study was conducted to evaluate an insecticidal formulation containing two active ingredients, i.e., thiamethoxam plus chlorantraniliprole, when applied on grains and different surfaces against adults of the aforementioned species. Complete mortality was observed for *S. oryzae* on all three commodities, for *R. dominica* on wheat and rice, and for *T. castaneum* on wheat, 14 days post-initial exposure at the highest dose. At the end of the 90-day storage period, *S. oryzae* showed the maximum mortality, whereas *T. granarium* showed the lowest. Bioassays on surfaces revealed that thiamethoxam plus chlorantraniliprole applied on metal killed more adult individuals of all species than on cement, ceramic, or wood. *Sitophilus oryzae* was found to be the most sensitive on metal, followed by *R. dominica*, *T. castaneum*, and *T. granarium*.

**Abstract:**

An insecticide containing the neonicotinoid thiamethoxam + the diamide chlorantraniliprole was evaluated against adults of *Rhyzopertha dominica*, *Tribolium castaneum*, *Trogoderma granarium*, and *Sitophilus oryzae* under laboratory bioassays both on freshly treated grain as well as on treated grain stored over 90 days for its persistence in efficacy. In laboratory bioassays, the insecticide was applied on wheat, maize, or rice at four doses, while in persistence bioassays on wheat at the same doses. Mortality and progeny were assessed in both laboratory and persistence bioassays. After 14 days of exposure, *S. oryzae* exhibited 100% mortality on all three commodities at the highest dose, while *R. dominica* showed complete mortality on wheat or rice and *T. castaneum* on wheat. For a period of 90 days, *S. oryzae* exhibited 42.69% mortality, followed by *R. dominica* (35.26%), *T. castaneum* (27.08%), and *T. granarium* (18.63%) at the highest dose. Progeny was successfully suppressed in all cases of complete mortality in laboratory bioassays and for *S. oryzae* for 90 days in persistence bioassays. Laboratory trials were also performed on plywood, concrete, ceramic tile, and steel at one dose. The highest mortality was observed on steel, followed by concrete, ceramic tile, and plywood for all insect species tested. This study demonstrates that thiamethoxam + chlorantraniliprole is effective against the tested species depending on exposure, storage period, surface, commodity, and dose.

## 1. Introduction

Globally, more than 1663 insect species are recognized as stored-grain pests [1]. Among them, *Rhyzopertha dominica* (F.) (Coleoptera: Bostrychidae) is one of the most devastating primary pest species of rice, cornbread, cereals, and wheat, among other commodities across the world [1,2]. Larvae and adults of the secondary pest *Tribolium castaneum* (Herbst) (Coleoptera: Tenebrionidae) are voracious feeders that can cause significant damage to a broad range of grains, milled cereal products, and flours worldwide, infesting up to 233 types of commodities [1,3]. *Trogoderma granarium* Everts (Coleoptera: Dermestidae) is classified as a quarantine species in several countries of Africa, America, Europe, and Oceania, according to the European and Mediterranean Plant Protection Organization [4]. *Sitophilus oryzae* (L.) (Curculionidae: Coleoptera) is a cosmopolitan primary pest, causing degradation in both the quality and quantity of grains [1,5]. Insecticides, particularly pyrethroids and organophosphates, have been extensively employed as protectants against stored-grain insects [6]. However, the frequent and excessive application of these insecticides has resulted in resistance emergence in stored-product pests, which has diminished the effectiveness of pest management strategies [6,7,8,9]. The approaches to address the problem of resistance in stored-grain pests towards a particular class of pesticides involve reducing the excessive use of these insecticides and/or using an alternative insecticide group that has a distinct mode of action [10,11,12]. The combined implementation of insecticides with distinct modes of action to manage pests may potentially demonstrate higher efficacy when compared to the individual application of insecticides. For instance, the combined application of spinosad (spinosyn) and chlorpyrifos-methyl (organophosphate) provided a long-lasting effect against four psocid pest species of stored products, while the solitary application was less effective [13]. The combination of the diatomaceous earth silicoSec with spinosad was more efficient than every compound alone in controlling *Tribolium confusum* Jacquelin du Val (Coleoptera: Tenebrionidae) [14]. In the same study, however, the combined application of both insecticides did not result in significantly elevated mortality against *S. oryzae* compared to single applications of each treatment. Gad et al. [15], who studied the efficacy of different combinations of spinosad with three chitin synthesis inhibitors, chlorfluazuron, hexaflumuron, or lufenuron, on stored wheat against *S. oryzae*, exhibited that combined applications resulted in higher mortality rates and complete progeny inhibition compared to single treatments of each insecticide. 

Neonicotinoids are systemic compounds that have broad-spectrum activity against various pests, such as sucking insects (e.g., leafhoppers, whiteflies, and aphids), as well as several flies and moths [16]. They demonstrate high efficacy even at low doses, with low toxicity to vertebrates, including humans [17,18]. The nicotinic acetylcholine receptors (nAChRs) play a crucial role in the rapid neurotransmission, learning, and memory processes in insects [19,20]. Neonicotinoids function by acting as agonists of nicotinic acetylcholine receptors, resulting in the overstimulation of the cholinergic synapse and insect mortality [21,22]. These insecticides have been used since the 1990s for crop protection against insect pests; however, enhanced resistance has been well documented, resulting from their excessive use [23]. Diamides have gained significant attention in the market due to their broad insecticidal spectrum, high efficiency, and highly favorable ecological, biological, and toxicological properties [24,25,26]. Diamide insecticides are relatively safe for beneficial organisms of insects and are categorized as minimally detrimental to benign according to the categorization established by the International Organization for Biological Control (IOBC) for non-target organisms [27]. For example, flubendiamide, a phthalic diamide that targets mostly Lepidoptera, while chlorantraniliprole and cyantraniliprole are anthranilic diamides with a wider spectrum of activity [25]. Chlorantraniliprole and cyantraniliprole have been demonstrated to effectively control a variety of insect species of agricultural importance that belong to numerous insect orders [28,29,30]. Diamide insecticides are employed for the control of pests through selective activation of the ryanodine receptors of the insect muscle cells, inducing continuous muscular contraction, vomiting symptoms, paralysis, feeding inhibition, and ultimately succumbing of the insect to mortality [31,32]. Nevertheless, the utilization of diamides to a great extent during the past decade has led to the emergence of resistance in several of the world’s most harmful lepidopteran species [33].

The two active ingredients (a.i.), thiamethoxam and chlorantraniliprole, exhibit different mechanisms of action acting on distinct physiological pathways in insects. These modes of action give each insecticide its own specific way of controlling pest populations. The combination of thiamethoxam and chlorantraniliprole has proven efficient against a range of serious field pests [34,35]. However, it has never been evaluated against stored-grain species. Considering that the efficacy of insecticides may differ depending on several factors, including the grain type, the application surface, and the target organism [36,37,38], this study aims to examine the efficacy of chlorantraniliprole + thiamethoxam against four major pests of stored grains, *S. oryzae*, *T. castaneum*, *T. granarium*, and *R. dominica.* The assessments are conducted on wheat, maize, and rice, and concrete, steel, plywood, and ceramic tile.

## 2. Materials and Methods

### 2.1. Grains

The grains used in this study were wheat, *Triticum aestivum* L. (var. Noor 2013), rice, *Oryza sativa* L. (var. Kainat Basmati), and maize, *Zea mays* L. (var. DK-6525). All commodities were free from pest infestations, pesticides, and impurities. Moisture contents of rice, maize, and wheat were measured using a standardized Dickey-John moisture meter (Dickey-John Multigrain CAC II, Dickey-John Co., Auburn, IL, USA) and measured at 11.2%, 10.5%, and 11.5%, respectively.

### 2.2. Insect Culture

The populations of *S. oryzae*, *T. granarium*, *T. castaneum*, and *R. dominica* used in this study were acquired from the Microbial Control Laboratory, University of Agriculture, Faisalabad. These insect populations had been cultured for over 10 years and deprived of any exposure to insecticides. Adults of mixed sex and <24 h old were used for *T. granarium.* For *T. castaneum*, *R. dominica*, and *S. oryzae*, adults of mixed sex < 2 weeks old were used. All insects were reared at 30 °C, 24 h complete darkness, and 65% RH on sound wheat, except *T. castaneum*, which was reared on the wheat flour along with 5% brewer’s yeast at 65% humidity, 30 °C temperature and complete absence of light of 24 h [39].

### 2.3. Formulation

VoliamFlexi 300 SC (Syngenta, Pakistan), the suspension concentrate, containing 200 g/L thiamethoxam a.i. and 100 g/L chlorantraniliprole a.i., was used in the laboratory, persistence bioassays, and surface treatment bioassays.

### 2.4. Laboratory Bioassays

Despite the fact that VoliamFlexi is not registered for application on grains, the range of doses of thiamethoxam and chlorantraniliprole has been previously tested [40,41]. The insecticide was applied as an aqueous solution of 1 mL at four doses, (0.01 mg thiamethoxam + 0.005 mg chlorantraniliprole)/kg grain (0.01 ppm + 0.005 ppm, respectively) (=G1), (0.1 mg thiamethoxam + 0.05 mg chlorantraniliprole)/kg grain (0.1 ppm + 0.05 ppm, respectively) (=G2), (1 mg thiamethoxam + 0.5 mg chlorantraniliprole)/kg grain (1 ppm + 0.5 ppm, respectively) (=G3), and (5 mg thiamethoxam + 2.5 mg chlorantraniliprole)/kg grain (5 ppm + 2.5 ppm, respectively) (=G4), on each of the three different commodities: wheat, rice, and maize. Spraying was carried out by applying 1 mL of aqueous solution containing the appropriate volume of the insecticide congruent to each dose on separate trays containing 1 kg of each commodity. The process of spraying was performed using an airbrush (Master Multipurpose Airbrush, San Diego, CA, USA). One kg of maize, wheat, or rice was sprayed with 1 mL of distilled water, serving as the control group, with a separate airbrush kept for spraying controls only. Separate trays were used for each dose of the insecticide and each commodity tested. Following the completion of the treatment, all grains were transferred into 5 L glass jars and subjected to manual shaking for a minimum of 10 min to ensure uniform distribution of the insecticide throughout the entire mass of grains [42]. Subsequently, 3 samples of 20 g were extracted from each untreated and treated lot and introduced into glass vials (125 mm height and 75 mm diameter). To maintain sample integrity and prevent cross-contamination, a separate scoop was used for each individual glass jar during the sampling process. Each sample was weighted with an ELB 300 electronic balance (Shimadzu, Kyoto, Japan). A new thin layer was utilized each time of weighing. In the middle of the closure of each glass vial, a hole of 15 mm diameter, which was properly overlayed with gauze, served for the aeration inside the vial. Afterward, 50 adults of mixed sex of each species tested were placed inside each glass vial. To restrain the insects from escaping, the internal neck of all glass vials was well covered with polytetrafluoroethylene (Sigma-Aldrich Chemie GmbH, Taufkirchen, Germany). All glass vials were kept inside incubators set at 65% RH and 30 °C. Mortalities of adults were assessed after 7 and 14 days of exposure by prodding the individuals with the help of a brush under a Leica stereomicroscope (Wild M3B, Heerbrugg, Switzerland) to provoke movement. Different vials were used for each exposure interval. The progeny production of the tested adults of each pest species was also evaluated for each treated commodity. Thus, after 14 days of exposure, alive and dead parental individuals were discarded from vials. Subsequently, vials were placed inside an incubator under the aforementioned conditions to record the number of progeny individuals/vial after 62 days for *T. castaneum* and *R. dominica*, 60 days for *S. oryzae*, and after 46 days for *T. granarium* [39,43]. For *T. castaneum* and *T. granarium*, adults and immatures were recorded as progeny, whereas for *S. oryzae* and *R. dominica*, only adults were recorded as progeny since immatures of these species develop inside the kernels. The entire procedure was replicated one more time (twice in total) with the implementation of new insects, commodity lots, and vials each time.

### 2.5. Persistence Bioassays

After a 7-day exposure, the effects of the treatment at four doses on wheat along with controls were evaluated for 3 months in 4 laboratory trials every 30 days (0, 30, 60, and 90 days) at 65% RH and 30 °C as described above. During the entire experimental period, the treated lots containing 1 kg of wheat were kept inside jars at the same humidity and temperature conditions as above, inside a chamber [39,44,45]. Mortalities of all insects were examined after 7 days of exposure to the insecticide, and progeny production was assessed as described above. In these bioassays, the mortality of parental individuals and progeny of *T. castaneum*, *R. dominica*, *S. oryzae*, and *T. granarium* was evaluated.

### 2.6. Surface Treatment Bioassays

Four surface types were employed: 2 cm thick concrete (D.G. Khan Cement, Lahore, Pakistan), 4 mm thick plywood (Virgin Wood Enterprises, Lahore, Pakistan), 1 mm thick steel (Pakistan Steel Mills Corporation, Karachi, Pakistan), and 8 mm thick ceramic tile (Shabbir Tiles & Ceramics Limited, Karachi, Pakistan). Concrete surfaces were created through the amalgamation of 1 kg of cement with 260 mL of tap water. Subsequently, approx. 20 g of the mixture was carefully positioned at the base of plastic boxes (10 cm × cm surface). The boxes were permitted to dry at 30 °C for 48 h. Plywood, steel, and ceramic tile surfaces were cut to the appropriate dimensions to fit properly into the boxes. Polytetrafluoroethylene dispersion (Sigma-Aldrich Chemie GmbH, Taufkirchen, Germany) was applied to the internal walls of the boxes to prevent insect escape. Three sub-replicates of each surface type were created. The aqueous solution of 1 mL VoliamFlexi, containing 0.05 mg thiamethoxam/cm^2^ + 0.025 mg chlorantraniliprole/cm^2^, was applied in the form of fine mist using an airbrush (Master Multipurpose Airbrush, USA) on the four different surfaces as aforementioned. The tested dose of the insecticidal formulation falls within the ranges of both a.i. that have been previously tested for surfaces [46,47]. A separate series of boxes were sprayed only with water to serve as control using a different airbrush. Separate boxes were prepared for each exposure interval. The treated surfaces were left to dry for one day, and subsequently, 0.2 g of wheat was added to the boxes. Then, 50 adults of each tested species were introduced to the boxes and placed inside an incubator set at 30 °C and 65% RH in darkness. Mortalities were assessed after 1, 2, 3, and 5 days. The entire procedure above was repeated once more (twice in total), using new boxes, wheat, and insects for each replication.

### 2.7. Statistical Analysis

Given the low mortality rates (<5%) observed in the control treatments for all insect species, it was determined that no correction was required for the recorded mortality values [48]. For the laboratory and persistence trials, data on mortality were corrected using Abbott’s equation [49] and, subsequently, log (x + 1)-transformed to ensure normal variance before analysis [50,51]. In both cases, a three-way analysis of variance (ANOVA) was performed on the data, with dose rate, exposure intervals, and commodities as the main effects and mortality as the response variable. Additionally, interactions among all main effects were considered. Progeny analysis in laboratory and persistence bioassays involved the use of three-way ANOVA, with dose, commodity, and insect species as the main effects and the number of individuals as the response variable. Interactions among main effects were also included in the analysis. To compare means of progeny and mortality, the Tukey–Kramer (HSD) test at *p* = 0.05 was employed [52]. For surface bioassays, surface mortality was corrected using Abbott’s equation [49]. The surface data were log (x + 1)-transformed before analysis to ensure normal variance [50,51]. A two-way ANOVA was performed, with treatment and time interval as the main effects and mortality as the response variable. Interactions among main effects were also included in the analysis. To compare means of mortality, the Tukey–Kramer HSD test at *p* = 0.05 was applied [52].

## 3. Results

### 3.1. Adult Mortality in Laboratory Bioassays

All main effects and the dose × exposure interaction were significant for all the species tested. The interaction commodity × dose was significant only for *T. granarium* and *T. castaneum*, while the commodity × exposure × dose interaction was significant only for *S. oryzae* and *R. dominica* (Table 1).

For all insect species tested, mortalities were higher in wheat, followed by rice and maize, regardless of the dose applied and the exposure interval (Table 2). However, mortality increased with the dose increment. The highest susceptibility was exhibited for *S. oryzae*, which demonstrated complete mortality after a 14-day exposure on all commodities tested at the highest dose. For *R. dominica*, the highest dose caused complete mortality on wheat and rice after 14 days of exposure, while *T. castaneum* exhibited complete mortality only on wheat. The lowest susceptibility was observed for *T. granarium*, which reached 92.79% mortality on wheat at the highest dose.

### 3.2. Progeny Production in Laboratory Bioassays

Concerning progeny, all main effects and their interactions were significant (Table 3). Suppressed progeny was observed for *S. oryzae* in wheat treated with the insecticide at G2 and in all commodities at G3 and G4 (Table 4). For *T. castaneum*, the progeny suppression was observed on wheat and rice at the highest dose, while on maize, the progeny reached a mean of 7 individuals. In *T. granarium*, the progeny was reduced but not suppressed. The lowest progeny production was observed for the highest dose of the insecticide on wheat, followed by rice and maize. For *R. dominica*, complete progeny suppression was observed in wheat and rice at G3 and in all commodities at G4. Overall, thiamethoxam + chlorantraniliprole was more effective in the reduction of progeny production for *S. oryzae*, followed by *R. dominica*, *T. castaneum*, and *T. granarium*.

### 3.3. Adult Mortality in Persistence Bioassays

After 7 days of exposure, the main effects and associated interaction were significant for all tested pest species (Table 5). All insect species exhibited higher mortalities as the dose increased in all storage periods (Table 6). Complete mortality was observed for *S. oryzae*, *R. dominica*, and *T. castaneum* on the 0 day of the trial at the highest dose. However, the efficacy of thiamethoxam + chlorantraniliprole decreased with the increment of the storage period for all species, regardless of the dose used.

### 3.4. Progeny Production in Persistence Bioassays

Regarding progeny, all main effects and associated interactions significantly affected offspring production (Table 7). Suppressed progeny was observed for *S. oryzae* at the 0 day of the trial at all implemented doses (Table 8). After 90 days, the highest dose of the treatment successfully suppressed offspring production in *S. oryzae*. For *T. castaneum*, the progeny was suppressed at G3 and G4 on the 0 day of the trial. However, after 90 days offspring ranged from a mean of 14.41 to 42.76 individuals/vial. Regarding *T. granarium*, progeny was suppressed only at the highest dose on the 0 day of the trial, while after 90 days, the mean offspring ranged from 21.86 to 53.15 mean individuals/vial. No progeny was observed for *R. dominica* at the 0 day of the trial for G2, G3, and G4. After 90 days, progeny ranged from 9.73 to 37.78 mean individuals/vial depending on the dose applied. Overall, offspring was higher for *T. granarium*, followed by *T. castaneum*, *R. dominica*, and *S. oryzae*.

### 3.5. Adult Mortality in Surface Treatment Bioassays

All main effects significantly affected mortality in all tested pest species (Table 9). However, the associated interaction of surface × exposure only affected *T. castaneum* and *R. dominica* significantly. Significantly higher mortality was exhibited on steel for all tested insect species, followed by concrete, ceramic tile, and plywood at 2, 3, and 5 days after exposure (Table 10). Overall, *S. oryzae* and *R. dominica* were more susceptible to the treatment on all tested surfaces compared to *T. castaneum* and *T. granarium*. Mortality increased with the increase of all exposure periods for all tested insect species.

## 4. Discussion

This study examined the efficacy of chlorantraniliprole + thiamethoxam as wheat, maize, and rice protectants and as structural (concrete, steel, plywood, and ceramic tile) treatments against one secondary and three primary pests of stored grains in laboratory and persistence trials. *Sitophilus oryzae* and *R. dominica* demonstrated higher mortalities and lower progeny than *T. granarium* and *T. castaneum* in both laboratory and persistence bioassays. Previously, numerous insecticides have been assessed for their efficacy as grain protectants, highlighting the variation in efficacy depending on the target insect species/strain. For instance, great variation in the efficacy of commercial diatomaceous earths (DEs) was observed among several insect species, such as *Sitophilus zeamais* (Motschulsky) (Coleoptera: Curculionidae) and *Prostephanus truncatus* (Horn) (Coleoptera: Bostrychidae) [38]. Spinosad demonstrated higher efficacy against *R. dominica* compared to its effectiveness against *S. oryzae* when applied to grains in both dry and liquid forms [53], as also exhibited in previous research [36]. Daglish and Nayak [54] reported that *Cryptolestes ferrugineus* (Stephens) (Coleoptera: Laemophloeidae) exhibited 100% mortality when treated with the oxadiazine, indoxacarb on wheat after 14 days of exposure, while no effect was observed on the mortality of *T. castaneum*. Chlorantraniliprole exhibited higher efficacy against *R. dominica*, *S. oryzae*, *Liposcelis bostrychophila* Badonnel (Psocoptera: Liposcelididae), and *Ephestia kuehniella* Zeller (Lepidoptera: Pyralidae), compared to *T. confusum* [55]. On the contrary, *E. kuehniella* larvae exhibited lower mortality compared to *T. confusum* larvae when treated with deltamethrin, alpha-cypermethrin, thiamethoxam, and pirimiphos-methyl [46]. Rigaux et al. [56], who studied 14 *T. castaneum* strains, observed varying susceptibility levels to DE after a 7-day exposure, while Arnaud et al. [57], who exposed seven *T. castaneum* strains to four commercially available DEs, later confirmed the variation in susceptibility among strains of this species. The combination of *Beauveria bassiana* (Balsamo-Crivelli), Vuillemin (Hypocreales: Cordycipitaceae), and spinetoram caused higher immediate mortality for *R. dominica* and *Sitophilus granarius* (L.) (Coleoptera: Curculionidae) compared to *T. castaneum* and *T. granarium* [45].

An additional determinant influencing the performance of grain protectants lies in the specific commodity to which they are applied. In this study, thiamethoxam + chlorantraniliprole demonstrated higher efficacy on wheat, with rice and maize following, against all pest species in the laboratory bioassays. The dependence of insecticide efficacy on the type of commodity has become evident in the literature. *Rhyzopertha dominica* exhibited higher mortality in wheat compared to rice and maize when treated with thiamethoxam and silicoSec, alone or in combination [40]. In terms of efficacy, chlorantraniliprole displayed lower efficacy in whole rice and maize compared to wheat, peeled rice, barley, and oats against *L. bostrychophila*. Conversely, for *T. confusum* larvae, chlorantraniliprole exhibited higher effectiveness in maize compared to other commodities [55]. Mortalities of *S. oryzae*, *T. confusum*, and *C. ferrugineus* were higher on barley or wheat compared to rice and maize when treated with DE and the plant extract bitterbarkomycin (BBM) [58]. The residual efficacy of spinosad against *Oryzaephilus surinamensis* (L.) (Coleoptera: Silvanidae), *T. granarium*, and *T. castaneum* was higher on oats, with wheat, maize, and rice following [59]. In addition to grain type, the efficacy of grain protectants is influenced by the class of the commodity. For instance, Fang et al. [36], who studied the performance of spinosad on four wheat classes (durum, hard red winter, soft red winter, and hard red spring wheat) against adults of *O. surinamensis*, *T. castaneum*, *R. dominica*, and *S. oryzae*, illustrated substantial differences in pests’ mortalities in different commodity classes. Physicochemical properties of the kernels, such as weight, shape, size, porosity, bulk density, protein content, and gluten index, are important factors that can impact the residue coverage of grain protectants, thereby influencing their efficacy across different grain commodities [60].

Different concentrations of a.i. in grain protectants result in varying levels of persistence. For instance, when wheat was treated with alpha-cypermethrin at 0.125 ppm and 0.250 ppm, deltamethrin at 0.125 ppm and 0.250 ppm, beta-cyfluthrin at 0.125 ppm and 0.250 ppm, the mortality rates of *T. confusum* adults after 172 days of storage and 7 days after exposure were 8.8%, 48.1%, 12.7%, 27.7%, 44.4%, and 77.7%, respectively [44]. Furthermore, several pests exhibit a general trend of tolerance to various insecticides in persistence trials. In the current study, *S. oryzae* demonstrated the highest susceptibility in persistence trials, followed by *R. dominica*, *T. castaneum*, and *T. granarium*. In line with this study, previous research has demonstrated that *T. granarium* and *T. castaneum* were the least susceptible during persistence trials compared to other major stored grain pests. Wakil et al. [45] reported that the combination of *B. bassiana* and spinetoram caused higher mortality for *R. dominica* and *S. granarius* compared to *T. granarium* and *T. castaneum* after a 180-day storage period. The low susceptibility of *T. granarium* and *T. castaneum* in persistence trials compared to *R. dominica* and *S. granarius* has also been exhibited when exposed to fipronil and *B. bassiana* after a storage period of 180 days [39].

In addition to parental mortality, a grain protectant deemed effective should demonstrate the capacity to inhibit offspring production within the treated substrate. In both laboratory and persistence bioassays of the current study, thiamethoxam + chlorantraniliprole demonstrated successful suppression of offspring, even in cases where complete parental mortality was not attained. Previous research has demonstrated that the application of two formulations of chlorantraniliprole at 10 ppm on maize caused 96.1–98.9% mortality in *P. truncatus* and suppressed progeny after 45 days [41]. Complete progeny suppression of two populations of *R. dominica* was achieved by the application of 0.75 ppm of thiamethoxam when mortality reached 93.1–100% on wheat and 93.6% on rice [40]. In the current study, the combined application of 1 ppm thiamethoxam + 0.5 ppm chlorantraniliprole and 5 ppm thiamethoxam + 2.5 ppm chlorantraniliprole caused progeny suppression of *R. dominica*, with mortalities reaching 68.50–74.29% and 94.86%, respectively. Suppressed progeny was also observed for *S. oryzae* and *T. castaneum*, even when parental mortality was moderate. Taking into account the aforementioned studies on the single application of thiamethoxam and chlorantraniliprole, the combined application of these a.i. can suppress progeny production at lower doses, even without exhibiting complete parental mortality. Considering also that complete suppression of progeny production does not always occur despite achieving 100% adult mortality [61], the combined effect of thiamethoxam + chlorantraniliprole on both direct adult mortality and offspring emergence indicates that this formulation can be regarded as an effective measure for long-term protection of wheat in storage. Nevertheless, the efficacy of this formulation requires further evaluation against various pest species and commodities.

Depending on the application surfaces, the efficacy of insecticides varies [37,62,63]. In developing countries, grain storage occurs in a range of structures, including granaries, mud/thatch rhombuses, platforms, cribs, earthen pots/baskets, binishells, storage bags made of jute, hessian, and polyethylene, and steel/concrete silos [64,65]. The interaction between an insecticide and the properties of the application surface involves the absorption of the insecticide into the surface substrate, which can influence its efficacy [62]. Typically, insecticidal residues exhibit greater persistence on non-porous surfaces such as tile and steel in comparison to more porous surfaces such as concrete or various types of wood [37,62]. To this end, research on the insect growth regulator (IGR) methoprene has demonstrated variations in its persistence on different surfaces [65,66]. This was later exhibited again by Arthur et al. [67], who studied the efficacy of two IGRs and two pyrethroid insecticides against *T. granarium* on various surfaces, highlighting the elevated efficacy of all treatments on metal surfaces. Through an investigation into the efficacy of malathion on various bag fabrics used for storage, Paudyal et al. [68] observed that polypropylene bags exhibited higher mortality rates and lower offspring emergence of *T. castaneum* compared to other absorbent fabric options. Our results demonstrate that thiamethoxam + chlorantraniliprole was considerably more efficient on non-porous surfaces, i.e., metal [69], followed by surfaces with higher porosity, i.e., concrete, ceramic tile, and plywood [37] against all tested storage pests. Nevertheless, this combination exhibited moderate insecticidal performance for all species.

## 5. Conclusions

Overall, the tested insecticidal formulation containing thiamethoxam and chlorantraniliprole has higher effectiveness in controlling *S. oryzae* and *R. dominica* on all tested grains but lower efficiency for *T. castaneum* and *T. granarium*. Concerning surface treatment, thiamethoxam + chlorantraniliprole demonstrates intermediate performance for all insect species. Our results indicate that the insecticide inhibits the reproductive capabilities of *S. oryzae*, *T. castaneum*, *T. granarium*, and *R. dominica*; however, its overall efficacy is influenced by the nature of the commodity, surface substrate, exposure interval, and application dose. Due to the limited number of registered grain protectants used in stored products, there is a growing demand for new and effective tools to enhance protection in storage facilities [12]. Our study has provided insights into the potential use of thiamethoxam + chlorantraniliprole in the storage environment, although it has not demonstrated broad-spectrum efficacy.

## Figures and Tables

**Table 1:** ANOVA parameters for adult mortality of *S. oryzae*, *T. castaneum*, *T. granarium*, and *R. dominica* on three commodities treated with four doses of thiamethoxam + chlorantraniliprole in laboratory trials (total df = 143).

		*S. oryzae*		*T. castaneum*		*T. granarium*		*R. dominica*	
Effect	df	F	*p*	F	*p*	F	*p*	F	*p*
Commodity	2	112.65	<0.01	93.94	<0.01	68.10	<0.01	118.11	<0.01
Exposure	1	625.98	<0.01	331.97	<0.01	483.97	<0.01	593.73	<0.01
Dose	3	1442.43	<0.01	1006.98	<0.01	1049.98	<0.01	1570.71	<0.01
Commodity × exposure	2	1.09	0.34	0.28	0.75	0.14	0.86	2.65	0.07
Commodity × dose	6	0.75	0.61	2.80	0.01	2.18	0.04	1.44	0.20
Dose × exposure	3	6.37	<0.01	43.40	<0.01	102.48	<0.01	41.38	<0.01
Commodity × exposure × dose	6	8.89	<0.01	1.37	0.23	0.24	0.96	9.24	<0.01

**Table 2:** Mean mortality (% ± SE) of *S. oryzae*, *T. castaneum*, *T. granarium*, and *R. dominica* adults after a 7- and 14-day exposure to four doses of 0.01 ppm + 0.005 ppm (=G1), 0.1 ppm + 0.05 ppm (=G2), 1 ppm + 0.5 ppm (=G3), and 5 ppm + 2.5 ppm (=G4) of thiamethoxam + chlorantraniliprole, respectively, on three grain commodities, in laboratory trials. For each species, within each column, means followed by the same uppercase letter are not significantly different (in all cases, df = 2, 17, Tukey–Kramer (HSD) test at *p* = 0.05). For each species, within each row, means followed by the same lowercase letter are not significantly different (in all cases, df = 3, 23, Tukey–Kramer (HSD) test at *p* = 0.05).

		Exposure Interval (7 Days)					
		Dose					
Species	Commodity	G1	G2	G3	G4	F	*p*
*S. oryzae*	Wheat	26.74 ± 1.47 Ad	44.03 ± 1.74 Ac	67.75 ± 2.06 Ab	90.81 ± 1.58 Aa	260	<0.01
	Maize	18.55 ± 1.58 Bd	33.34 ± 1.19 Bc	54.30 ± 1.87 Bb	68.04 ± 1.56 Ca	195	<0.01
	Rice	21.18 ± 2.08 ABd	38.98 ± 2.06 ABc	60.21 ± 2.65 ABb	75.66 ± 1.98 Ba	116	<0.01
	F	5.81	9.80	9.20	45.2		
	*p*	0.01	<0.01	<0.01	<0.01		
*T. castaneum*	Wheat	17.60 ± 2.21 Ad	35.65 ± 1.93 Ac	51.32 ± 1.77 Ab	74.46 ± 1.64 Aa	160	<0.01
	Maize	9.17 ± 2.04 Bd	22.14 ± 1.97 Bc	34.77 ± 1.49 Cb	53.57 ± 1.81 Ca	105	<0.01
	Rice	13.06 ± 1.57 ABd	26.45 ± 2.68 Bc	42.97 ± 1.65 Bb	61.85 ± 1.56 Ba	120	<0.01
	F	4.26	9.60	25.3	39.4		
	*p*	0.02	<0.01	<0.01	<0.01		
*T. granarium*	Wheat	13.98 ± 1.68 Ad	28.39 ± 1.39 Ac	36.95 ± 1.49 Ab	57.16 ± 1.38 Aa	146	<0.01
	Maize	7.49 ± 0.82 Bd	19.78 ± 1.94 Bc	27.98 ± 1.94 Bb	41.66 ± 1.29 Ca	83.2	<0.01
	Rice	10.47 ± 1.86 ABd	23.39 ± 2.05 ABc	32.54 ± 2.53 ABb	48.59 ± 1.47 Ba	63.1	<0.01
	F	4.54	5.62	4.86	31.3		
	*p*	0.02	0.01	0.02	<0.01		
*R. dominica*	Wheat	20.77 ± 1.30 Ad	38.45 ± 0.96 Ac	54.06 ± 1.62 Ab	85.74 ± 1.92 Aa	339	<0.01
	Maize	13.67 ± 1.33 Bd	27.03 ± 1.01 Bc	40.70 ± 2.15 Bb	58.50 ± 2.13 Ca	122	<0.01
	Rice	16.83 ± 1.54 ABd	34.68 ± 1.40 Ac	52.24 ± 1.71 Ab	67.65 ± 1.94 Ba	173	<0.01
	F	6.44	25.8	15.4	47.8		
	*p*	<0.01	<0.01	<0.01	<0.01		
		Exposure interval (14 days)					
*S. oryzae*	Wheat	42.80 ± 1.20 Ad	63.69 ± 1.80 Ac	86.28 ± 1.82 Ab	100.00 ± 0.00 a	316	<0.01
	Maize	29.64 ± 2.46 Bd	45.16 ± 1.60 Cc	71.72 ± 1.37 Cb	100.00 ± 0.00 a	364	<0.01
	Rice	36.55 ± 1.25 Ad	52.43 ± 1.87 Bc	78.97 ± 1.93 Bb	100.00 ± 0.00 a	358	<0.01
	F	14.3	28.0	17.8			
	*p*	<0.01	<0.01	<0.01			
*T. castaneum*	Wheat	21.99 ± 1.89 Ad	47.05 ± 2.70 Ac	68.02 ± 2.79 Ab	100.00 ± 0.00 Aa	233	<0.01
	Maize	15.41 ± 1.82 Bd	32.13 ± 3.15 Bc	46.54 ± 2.10 Bb	89.75 ± 1.96 Ba	188	<0.01
	Rice	18.32 ± 0.9 ABd	38.74 ± 1.54 ABc	55.70 ± 2.62 Bb	93.50 ± 2.95 ABa	216	<0.01
	F	4.20	8.54	18.2	6.41		
	*p*	0.03	<0.01	<0.01	<0.01		
*T. granarium*	Wheat	19.24 ± 1.02 Ad	36.06 ± 2.02 Ac	51.17 ± 1.91 Ab	92.79 ± 1.56 Aa	352	<0.01
	Maize	11.62 ± 1.21 Bd	28.09 ± 2.23 Bc	39.36 ± 1.59 Bb	78.45 ± 1.46 Ca	290	<0.01
	Rice	14.66 ± 1.97 ABd	31.12 ± 1.76 ABc	47.91 ± 2.63 Ab	85.64 ± 1.13 Ba	242	<0.01
	F	6.87	3.98	8.48	26.2		
	*p*	<0.01	0.04	<0.01	<0.01		
*R. dominica*	Wheat	33.18 ± 1.82 Ad	51.67 ± 1.75 Ac	74.29 ± 1.61 Ab	100.00 ± 0.00 Aa	370	<0.01
	Maize	18.91 ± 1.66 Cd	37.46 ± 2.46 Bc	63.91 ± 1.56 Bb	94.86 ± 1.55 Ba	318	<0.01
	Rice	25.24 ± 1.30 Bd	42.53 ± 2.22 Bc	68.50 ± 2.45 ABb	100.00 ± 0.00 Aa	334	<0.01
	F	19.6	11.0	7.33	10.8		
	*p*	<0.01	<0.01	<0.01	<0.01		

**Table 3:** ANOVA parameters for progeny production of *S. oryzae*, *T. castaneum*, *T. granarium*, and *R. dominica* on three commodities treated with four doses of thiamethoxam + chlorantraniliprole in laboratory trials (total df = 359).

Effect	df	F	*p*
Dose	4	6706.36	<0.01
Commodity	2	17.46	<0.01
Species	3	402.61	<0.01
Dose × commodity	8	51.97	<0.01
Dose × species	12	64.79	<0.01
Commodity × species	6	63.04	<0.01
Commodity × species × dose	24	36.09	<0.01

**Table 4:** Mean progeny number (±SE) of *S. oryzae*, *T. castaneum*, *T. granarium*, and *R. dominica* individuals/vial following a 60-, 62-, 46-, and 62-day exposure interval, respectively, on three commodities treated with four doses of 0.01 ppm + 0.005 ppm (=G1), 0.1 ppm + 0.05 ppm (=G2), 1 ppm + 0.5 ppm (=G3), and 5 ppm + 2.5 ppm (=G4) of thiamethoxam + chlorantraniliprole, respectively, in laboratory trials. For each species, within each column, means followed by the same uppercase letter are not significantly different (in all cases, df = 2, 17, Tukey–Kramer (HSD) test at *p* = 0.05). For each species, within each row, means followed by the same lowercase letter are not significantly different (in all cases, df = 4, 29, Tukey–Kramer (HSD) test at *p* = 0.05).

			Dose					
Species	Commodity	Control	G1	G2	G3	G4	F	*p*
*S. oryzae*	Wheat	84.73 ± 2.12 Ba	12.65 ± 1.62 Bb	0.00 Cc	0.00 c	0.00 c	949	<0.01
	Maize	95.06 ± 3.42 Ba	23.46 ± 1.43 Ab	18.40 ± 1.71 Ac	0.00 d	0.00 d	462	<0.01
	Rice	111.68 ± 3.70 Aa	21.28 ± 1.38 Ab	13.23 ± 1.45 Bc	0.00 d	0.00 d	620	<0.01
	F	18.5	14.8	53.3				
	*p*	<0.01	<0.01	<0.01				
*T. castaneum*	Wheat	93.06 ± 1.77 Aa	24.10 ± 2.04 Bb	17.06 ± 1.14 Bc	10.46 ± 1.26 Bd	0.00 Be	665	<0.01
	Maize	61.28 ± 1.53 Ba	31.96 ± 1.31 Ab	23.81 ± 1.85 Ac	16.45 ± 1.07 Ad	7.03 ± 1.30 Ae	206	<0.01
	Rice	55.65 ± 1.56 Ba	18.21 ± 0.89 Cb	15.68 ± 1.15 Bb	8.15 ± 1.44 Bc	0.00 Bd	344	<0.01
	F	153	21.3	9.34	11.4	29.2		
	*p*	<0.01	<0.01	<0.01	<0.01	<0.01		
*T. granarium*	Wheat	107.78 ± 2.48 Ba	35.31 ± 1.39 Bb	24.75 ± 1.83 Bc	18.30 ± 0.93 Bc	6.80 ± 1.16 Bd	585	<0.01
	Maize	118.33 ± 1.64 Aa	46.26 ± 1.49 Ab	31.40 ± 0.50 Ac	24.61 ± 1.86 Ad	17.61 ± 1.54 Ae	755	<0.01
	Rice	92.26 ± 1.49 Ca	37.18 ± 1.62 Bb	28.50 ± 1.11 ABc	21.95 ± 1.66 ABd	13.81 ± 1.24 Ae	462	<0.01
	F	46.5	15.1	6.82	3.98	17.0		
	*p*	<0.01	<0.01	<0.01	0.04	<0.01		
*R. dominica*	Wheat	116.40 ± 3.52 Aa	18.48 ± 1.41 Bb	11.56 ± 0.80 Bb	0.00 Bc	0.00 c	808	<0.01
	Maize	62.78 ± 1.37 Ca	24.76 ± 1.35 Ab	20.90 ± 1.50 Ab	9.16 ± 0.75 Ac	0.00 d	440	<0.01
	Rice	102.43 ± 2.48 Ba	26.93 ± 1.26 Ab	21.38 ± 1.62 Ab	0.00 Bc	0.00 c	854	<0.01
	F	113	10.6	16.5	149			
	*p*	<0.01	<0.01	<0.01	<0.01			

**Table 5:** ANOVA parameters for adult mortality of *S. oryzae*, *T. castaneum*, *T. granarium*, and *R. dominica* treated with four doses of thiamethoxam + chlorantraniliprole in laboratory persistence trials (total df = 95).

		*S. oryzae*		*T. castaneum*		*T. granarium*		*R. dominica*	
Effect	df	F	*p*	F	*p*	F	*p*	F	*p*
Dose	3	408.10	<0.01	329.31	<0.01	333.44	<0.01	363.46	<0.01
Storage period	3	809.13	<0.01	761.15	<0.01	528.58	<0.01	717.19	<0.01
Dose × storage period	9	15.34	<0.01	26.72	<0.01	29.40	<0.01	13.47	<0.01

**Table 6:** Mean mortality (% ± SE) of *S. oryzae*, *T. castaneum*, *T. granarium*, and *R. dominica* adults after a 7-day exposure to wheat treated with four doses of 0.01 ppm + 0.005 ppm (=G1), 0.1 ppm + 0.05 ppm (=G2), 1 ppm + 0.5 ppm (=G3), and 5 ppm + 2.5 ppm (=G4) of thiamethoxam + chlorantraniliprole, respectively, in four laboratory trials carried out from 0 to 90 days after treatment. For each species, within each column, means followed by the same uppercase letter are not significantly different (in all cases, df = 3, 23, Tukey–Kramer (HSD) test at *p* = 0.05). For each species, within each row, means followed by the same lowercase letter are not significantly different (in all cases, df = 3, 23, Tukey–Kramer (HSD) test at *p* = 0.05).

		Storage Period					
Species	Dose	0 Days	30 Days	60 Days	90 Days	F	*p*
*S. oryzae*	G1	54.45 ± 1.43 Ca	41.21 ± 1.70 Cb	34.53 ± 2.02 Dc	18.05 ± 1.28 Dd	85.3	<0.01
	G2	82.53 ± 1.58 Ba	57.00 ± 2.08 Bb	42.86 ± 1.76 Cc	24.63 ± 1.75 Cd	183	<0.01
	G3	96.92 ± 1.26 Aa	63.88 ± 1.72 Bb	51.87 ± 1.40 Bc	33.69 ± 0.92 Bd	383	<0.01
	G4	100.00 ± 0.00 Aa	84.51 ± 1.63 Ab	71.90 ± 1.78 Ac	42.69 ± 1.64 Ad	275	<0.01
	F	280	100	83.3	55.6		
	*p*	<0.01	<0.01	<0.01	<0.01		
*T. castaneum*	G1	37.75 ± 0.87 Da	28.31 ± 2.41 Db	21.59 ± 1.35 Db	9.09 ± 1.91 Cc	45.4	<0.01
	G2	65.98 ± 1.79 Ca	44.35 ± 1.36 Cb	28.73 ± 1.81 Cc	14.79 ± 1.49 BCd	182	<0.01
	G3	78.91 ± 1.79 Ba	56.31 ± 1.79 Bb	34.93 ± 1.30 Bc	21.65 ± 1.64 ABd	232	<0.01
	G4	100.00 ± 0.00 Aa	63.46 ± 1.33 Ab	45.89 ± 1.36 Ac	27.08 ± 2.21 Ad	453	<0.01
	F	374	74.6	48.9	16.9		
	*p*	<0.01	<0.01	<0.01	<0.01		
*T. granarium*	G1	25.92 ± 1.40 Da	16.80 ± 1.28 Cb	10.29 ± 1.66 Cc	0.00 ± 0.00 Dd	74.2	<0.01
	G2	42.98 ± 2.03 Ca	33.19 ± 1.70 Bb	18.54 ± 1.64 Bc	5.52 ± 1.99 Cd	78.7	<0.01
	G3	56.99 ± 1.18 Ba	38.33 ± 2.09 Bb	23.68 ± 2.18 Bc	12.42 ± 0.78 Bd	133	<0.01
	G4	92.12 ± 1.96 Aa	50.34 ± 1.62 Ab	37.78 ± 2.03 Ac	18.63 ± 1.71 Ad	286	<0.01
	F	278	66.9	37.0	35.0		
	*p*	<0.01	<0.01	<0.01	<0.01		
*R. dominica*	G1	47.61 ± 1.13 Da	36.16 ± 2.03 Db	28.66 ± 1.95 Cc	12.65 ± 1.68 Dd	71.1	<0.01
	G2	65.30 ± 1.74 Ca	51.87 ± 1.54 Cb	37.52 ± 1.72 Bc	20.87 ± 1.67 Cd	130	<0.01
	G3	83.33 ± 1.22 Ba	62.12 ± 1.51 Bb	43.66 ± 1.70 Bc	27.72 ± 1.32 Bd	272	<0.01
	G4	100.00 ± 0.00 Aa	73.03 ± 1.92 Ab	58.37 ± 1.37 Ac	35.26 ± 1.59 Ad	361	<0.01
	F	350	78.5	53.9	37.6		
	*p*	<0.01	<0.01	<0.01	<0.01		

**Table 7:** ANOVA parameters for progeny production of *S. oryzae*, *T. castaneum*, *T. granarium*, and *R. dominica* on wheat treated with four doses of thiamethoxam + chlorantraniliprole in laboratory persistence trials (total df = 119).

		*S. oryzae*		*T. castaneum*		*T. granarium*		*R. dominica*	
Effect	df	F	*p*	F	*p*	F	*p*	F	*p*
Dose	4	2931.37	<0.01	2713.96	<0.01	2021.81	<0.01	2995.36	<0.01
Storage period	3	141.89	<0.01	224.63	<0.01	252.93	<0.01	231.01	<0.01
Dose × storage period	12	19.46	<0.01	5.68	<0.01	2.18	0.01	15.29	<0.01

**Table 8:** Mean progeny number (±SE) of *S. oryzae*, *T. castaneum*, *T. granarium*, and *R. dominica* individuals/vial on wheat treated with four doses of 0.01 ppm + 0.005 ppm (=G1), 0.1 ppm + 0.05 ppm (=G2), 1 ppm + 0.5 ppm (=G3), and 5 ppm + 2.5 ppm (=G4) of thiamethoxam + chlorantraniliprole, respectively, in four laboratory trials carried out from 0 to 90 days after treatment. For each species, within each column, means followed by the same uppercase letter are not significantly different (in all cases, df = 4, 29, Tukey–Kramer (HSD) test at *p* = 0.05). For each species, within each row, means followed by the same lowercase letter are not significantly different (in all cases, df = 3, 23, Tukey–Kramer (HSD) test at *p* = 0.05).

		Storage Period					
Species	Dose	0 Days	30 Days	60 Days	90 Days	F	*p*
*S. oryzae*	0	79.08 ± 2.58 Ab	84.81 ± 2.44 Ab	98.63 ± 2.99 Aa	106.38 ± 1.93 Aa	24.7	<0.01
	G1	0.00 Bd	15.06 ± 1.52 Bc	24.15 ± 1.34 Bb	31.70 ± 2.08 Ba	87.7	<0.01
	G2	0.00 Bc	0.00 Cc	11.88 ± 1.47 Cb	19.78 ± 1.64 Ca	76.9	<0.01
	G3	0.00 Bb	0.00 Cb	0.00 Db	7.68 ± 1.52 Da	25.3	<0.01
	G4	0.00 B	0.00 C	0.00 D	0.00 E		
	F	934	819	659	694		
	*p*	<0.01	<0.01	<0.01	<0.01		
*T. castaneum*	0	98.63 ± 2.99 Ac	104.33 ± 3.18 Abc	112.93 ± 1.67 Ab	123.35 ± 2.09 Aa	17.6	<0.01
	G1	9.68 ± 1.50 Bd	27.90 ± 1.33 Bc	33.48 ± 1.39 Bb	42.76 ± 0.80 Ba	117	<0.01
	G2	1.16 ± 0.47 Cd	12.55 ± 1.43 Cc	21.76 ± 1.51 Cb	34.93 ± 2.56 Ca	73.2	<0.01
	G3	0.00 Cc	4.86 ± 1.39 Dc	16.96 ± 1.65 Cb	23.16 ± 1.54 Da	64.6	<0.01
	G4	0.00 Cc	0.00 Dc	5.48 ± 1.28 Db	14.41 ± 1.33 Ea	54.0	<0.01
	F	813	577	810	604		
	*p*	<0.01	<0.01	<0.01	<0.01		
*T. granarium*	0	91.03 ± 1.65 Ab	97.66 ± 3.32 Ab	107.93 ± 3.24 Aa	116.83 ± 1.18 Aa	20.1	<0.01
	G1	17.01 ± 1.69 Bd	31.90 ± 1.45 Bc	38.16 ± 1.64 Bb	53.15 ± 1.50 Ba	90.1	<0.01
	G2	8.86 ± 1.67 Cd	18.13 ± 1.65 Cc	24.73 ± 1.43 Cb	40.25 ± 1.69 Ca	66.6	<0.01
	G3	2.43 ± 0.86 Dd	11.88 ± 1.47 Cb	19.41 ± 1.45 Cc	29.68 ± 1.51 Da	72.8	<0.01
	G4	0.00 Dc	3.46 ± 0.42 Dc	10.95 ± 1.45 Db	21.86 ± 1.39 Ea	88.5	<0.01
	F	794	391	392	668		
	*p*	<0.01	<0.01	<0.01	<0.01		
*R. dominica*	0	87.96 ± 1.51 Ad	95.58 ± 2.52 Ac	115.18 ± 1.75 Ab	128.35 ± 1.60 Aa	94.6	<0.01
	G1	5.83 ± 2.96 Bc	19.80 ± 2.12 Bb	31.26 ± 2.06 Ba	37.78 ± 1.79 Ba	37.8	<0.01
	G2	0.00 Bd	6.03 ± 1.56 Cc	17.13 ± 1.69 Cb	25.81 ± 1.70 Ca	64.1	<0.01
	G3	0.00 Bb	0.00 Cb	10.75 ± 1.51 Ca	13.21 ± 1.61 Da	39.9	<0.01
	G4	0.00 Bb	0.00 Cb	0.00 Db	9.73 ± 1.54 Da	39.6	<0.01
	F	680	621	854	875		
	*p*	<0.01	<0.01	<0.01	<0.01		

**Table 9:** ANOVA parameters for adult mortality of *S. oryzae*, *T. castaneum*, *T. granarium*, and *R. dominica* treated with four doses of thiamethoxam + chlorantraniliprole in surface treatment trials (total df = 95).

		*S. oryzae*		*T. castaneum*		*T. granarium*		*R. dominica*	
Effect	df	F	*p*	F	*p*	F	*p*	F	*p*
Surface	3	302.73	<0.01	267.89	<0.01	198.43	<0.01	257.45	<0.01
Exposure	3	499.25	<0.01	267.03	<0.01	228.33	<0.01	335.21	<0.01
Surface × exposure	9	1.90	0.06	3.11	<0.01	1.49	0.16	2.61	0.01

**Table 10:** Mean mortality (% ± SE) of *S. oryzae*, *T. castaneum*, *T. granarium*, and *R. dominica* adults after a 1-, 2-, 3-, and 5-day exposure to four surface types treated with thiamethoxam + chlorantraniliprole at 0.05 mg thiamethoxam/cm^2^ + 0.025 mg chlorantraniliprole/cm^2^ in surface treatment trials. For each species, within each column, means followed by the same uppercase letter are not significantly different (in all cases, df = 3, 23, Tukey–Kramer (HSD) test at *p* = 0.05). For each species, within each row, means followed by the same lowercase letter are not significantly different (in all cases, df = 3, 23, Tukey–Kramer (HSD) test at *p* = 0.05).

		Surface Type					
Species	Exposure	Plywood	Ceramic Tile	Concrete	Steel	F	*p*
*S. oryzae*	1 day	21.01 ± 1.18 Dc	26.54 ± 1.61 Dc	38.96 ± 1.54 Db	47.25 ± 1.50 Da	65.2	<0.01
	2 days	28.35 ± 1.68 Cd	37.36 ± 0.98 Cc	49.15 ± 1.41 Cb	58.13 ± 1.67 Ca	79.5	<0.01
	3 days	39.85 ± 2.35 Bd	52.35 ± 2.27 Bc	65.65 ± 1.41 Bb	76.42 ± 1.24 Ba	70.7	<0.01
	5 days	57.18 ± 1.34 Ad	68.96 ± 1.33 Ac	76.30 ± 1.02 Ab	88.17 ± 1.36 Aa	104	<0.01
	F	85.8	129	149	158		
	*p*	<0.01	<0.01	<0.01	<0.01		
*T. castaneum*	1 day	11.65 ± 1.02 Dc	15.06 ± 1.14 Cc	23.97 ± 1.14 Db	32.86 ± 1.79 Da	52.5	<0.01
	2 days	17.51 ± 1.22 Cd	26.75 ± 1.93 Bc	36.71 ± 1.81 Cb	51.83 ± 1.78 Ca	73.4	<0.01
	3 days	24.50 ± 1.31 Bd	33.09 ± 2.35 Bc	47.22 ± 1.69 Bb	59.29 ± 2.42 Ba	58.7	<0.01
	5 days	36.30 ± 1.45 Ad	47.03 ± 1.68 Ac	58.43 ± 1.70 Ab	70.61 ± 1.12 Aa	95.3	<0.01
	F	69.0	52.7	83.2	74.1		
	*p*	<0.01	<0.01	<0.01	<0.01		
*T. granarium*	1 day	4.79 ± 1.37 Cd	9.89 ± 1.21 Dc	15.00 ± 0.98 Cb	26.62 ± 1.18 Da	60.8	<0.01
	2 days	10.24 ± 1.47 Cd	17.45 ± 1.33 Cc	28.41 ± 1.98 Bb	40.05 ± 1.97 Ca	57.5	<0.01
	3 days	18.53 ± 1.10 Bc	25.73 ± 1.43 Bc	36.05 ± 2.95 Bb	47.42 ± 1.53 Ba	44.0	<0.01
	5 days	29.62 ± 1.72 Ad	38.96 ± 1.60 Ac	49.28 ± 1.72 Ab	58.23 ± 2.11 Aa	47.5	<0.01
	F	56.7	78.3	49.5	58.1		
	*p*	<0.01	<0.01	<0.01	<0.01		
*R. dominica*	1 day	13.06 ± 1.04 Cc	18.53 ± 1.12 Dc	29.50 ± 2.23 Db	38.49 ± 1.31 Da	56.8	<0.01
	2 days	22.39 ± 1.75 Bd	32.07 ± 2.18 Cc	42.41 ± 1.17 Cb	57.23 ± 2.38 Ca	59.6	<0.01
	3 days	28.36 ± 2.33 Bd	41.16 ± 1.44 Bc	56.71 ± 1.41 Bb	68.84 ± 1.24 Ba	113	<0.01
	5 days	47.87 ± 1.48 Ad	58.24 ± 2.31 Ac	69.39 ± 1.97 Ab	79.09 ± 2.14 Aa	45.6	<0.01
	F	73.4	82.3	97.4	89.4		
	*p*	<0.01	<0.01	<0.01	<0.01		

## Data Availability

Data are available within the article.
